# Interaction of Mitochondria with the Endoplasmic Reticulum and Plasma Membrane in Calcium Homeostasis, Lipid Trafficking and Mitochondrial Structure

**DOI:** 10.3390/ijms18071576

**Published:** 2017-07-20

**Authors:** Jędrzej Szymański, Justyna Janikiewicz, Bernadeta Michalska, Paulina Patalas-Krawczyk, Mariasole Perrone, Wiesław Ziółkowski, Jerzy Duszyński, Paolo Pinton, Agnieszka Dobrzyń, Mariusz R. Więckowski

**Affiliations:** 1 Department of Biochemistry, Nencki Institute of Experimental Biology, Pasteur 3, 02-093 Warsaw, Poland; j.szymanski@nencki.gov.pl (J.S.); j.janikiewicz@nencki.gov.pl (J.J.); b.michalska@nencki.gov.pl (B.M.); p.patalas@nencki.gov.pl (P.P.-K.); j.duszynski@nencki.gov.pl (J.D.); a.dobrzyn@nencki.gov.pl (A.D.); 2 Department of Morphology, Surgery and Experimental Medicine, Section of Pathology, Oncology and Experimental Biology, Laboratory for Technologies of Advanced Therapies (LTTA), University of Ferrara, 44121 Ferrara, Italy; prrmsl@unife.it (M.P.); paolo.pinton@unife.it (P.P.); 3 Department of Bioenergetics and Nutrition, Gdańsk University of Physical Education and Sport, 80-336 Gdańsk, Poland; wiech@awf.gda.pl

**Keywords:** mitochondria, endoplasmic reticulum, plasma membrane, mitochondria-associated membranes (MAM), plasma membrane-associated membranes (PAM), lipid metabolism and trafficking

## Abstract

Studying organelles in isolation has been proven to be indispensable for deciphering the underlying mechanisms of molecular cell biology. However, observing organelles in intact cells with the use of microscopic techniques reveals a new set of different junctions and contact sites between them that contribute to the control and regulation of various cellular processes, such as calcium and lipid exchange or structural reorganization of the mitochondrial network. In recent years, many studies focused their attention on the structure and function of contacts between mitochondria and other organelles. From these studies, findings emerged showing that these contacts are involved in various processes, such as lipid synthesis and trafficking, modulation of mitochondrial morphology, endoplasmic reticulum (ER) stress, apoptosis, autophagy, inflammation and Ca${}^{2+}$ handling. In this review, we focused on the physical interactions of mitochondria with the endoplasmic reticulum and plasma membrane and summarized present knowledge regarding the role of mitochondria-associated membranes in calcium homeostasis and lipid metabolism.

## 1. Introduction

The close apposition of mitochondria to the endoplasmic reticulum (ER) and plasma membrane (PM) can be detected by different methods, although the transient interactions of the kiss-and-run mode might be difficult to capture. If interactions between organelles are strong enough, these close contacts can be isolated as interacting parts of the organelles. A recent paper by Giacomello and Pellegrini systemized the nomenclature related to the interaction between mitochondria and the ER [1]. The authors propose the use of the term mitochondria associated membranes (MAM) to describe material obtained from isolation/purification procedures of mitochondria-associated membranes and the term mitochondria-ER contacts (MERC) for architecture and ultrastructural organization. An isolated MAM fraction mostly consists of membrane fragments from the ER and the outer mitochondrial membrane (OMM) interacting at the time of isolation. More recently, the ER portion of the MAM fraction has also been regarded as a detergent-resistant lipid raft [2,3]. However, the term “mitochondria-associated membranes” does not narrow the focus down to only mitochondria and the ER because proteins from other cellular compartments have also been found in MAM, including proteins from the plasma membrane [4]. With respect to this observation, the contacts between mitochondria and the PM can also be assessed from the PM perspective. Physical interactions between the PM and intracellular membranes, including mitochondria, can be isolated as PM-associated membranes (PAM) [5]. In our review, we will not focus on the PAM fraction, and the reader interested in the topic can find more information about the molecular composition and function of PAM in [5,6,7]. The most recent literature summarizes protocols describing the isolation of different subcellular fractions, including: (a) mitochondria-associated membranes [8,9]; (b) ER-mitochondria encounter structure (ERMES) [10,11]; and (c) PAM [5].

## 2. Stay in Touch: Visualization of the Contacts between Mitochondria and Other Cellular Membranes

Contact sites between different organelles do not yet have a widely-accepted definition; nevertheless, naming several features that make these sites unique is advisable. One feature involves linkage across a contact site that should create a biochemically-distinct region, which is often achieved by excluding other organelles or protein complexes from that region. A second feature is the presence of specific molecular assemblies, which facilitate the exchange of material or signals in the contact site between organelles.

Mitochondria have been shown to form contact sites with several organelles. Examples of contact sites involve lipid droplets [12], peroxisomes, vacuoles [13] and the PM [14]. PM-mitochondria contacts are better studied in yeast, where proteins Num1 and Mdm36 have been shown to act as membrane anchors, which facilitate fission and enable uniform separation of mitochondria to daughter cells after cell division [15]. Some studies suggest that there is also a link between cell cortex localization and segregation of mitochondria during cell division in mammalian cells [16]. Mitochondria mainly participate in calcium signaling and ATP supply; however, there are estimates that almost 10% of the PM is covered with mitochondria [17], which may reflect the importance of such contact sites. Previous research has demonstrated that PAM are subcellular structures involved in store-operated Ca${}^{2+}$ (SOC) entry [7]. In particular, Orai1, a protein located in the PM, and stromal interaction molecule 1 (STIM1), an ER protein, were found in this fraction. Interaction of STIM1 aggregates with Orai1 results in activation of SOC channels, and close positioning of mitochondria to SOC channels reduces Ca${}^{2+}$-dependent channel inactivation. The most prominent examples are contact sites with the ER, which mark the site of mitochondrial fission [18] and are involved in the exchange of membrane lipids [19] and ions. The membranes of the ER and mitochondria at the contact sites are separated by 10–30 nm, which is beneficial for efficient transport between those structures, which is due to the reduction of the time required for the diffusional transport [20]. Therefore, the small gaps formed in the contact sites (in the range of tens of nanometers) enable direct metabolic channeling between organelles [20,21]. Investigation of isolated MAM fraction gives information about their protein and lipid composition, while different visualization methods can be used to characterize the structural and dynamic properties of the interorganelle contact sites in a quantitative way.

### 2.1. How Small Is a Contact Site? The Estimates from Electron Microscopy

Due to the development of many different types of electron microscopy (EM), studying the architecture of the organelles [22,23,24] and the interior of many cell types became possible. This technique enables visualization of the associations between mitochondria and other organelles, including ER [25]. Nonetheless, similar to many techniques, EM has limitations, such as the appearance of artifacts due to fixation. Hence, at the beginning, many micrographs were viewed with doubt [26]. A new method of sample preparation helped to avoid this problem seen in EM [27] and confirmed the link formed between the ER and mitochondria. The first observed signs of the close proximity of the ER to mitochondria were mentioned by Palade et al. in 1956 [23]; however, those findings did not capture much attention at that time. In 1959, Copeland and Dalton reported contact sites between mitochondria and the tubular form of the ER found in pseudo-branch gland cells [28]. A few years later, almost simultaneously, different groups observed and described the juxtaposition of the ER and OMM [9,29,30]. In accordance with data collected until the 1980s, a maximum of 80% of mitochondria were determined to be in contact with rough ER. Those findings were essential for subsequent discoveries in which the ER was identified to be the main calcium storage of the cell, a role previously attributed to mitochondria [31]. Collectively, these data undeniably show that mitochondria-ER contacts (MERCs) are one of the most widespread contacts among organelles in various cells [1].

Further development of the EM technique extended the use of EM to 3D imaging, providing access to novel information extracted from examined specimens. The analysis of high resolution images provided estimates of the size of gaps formed between adjacent membranes. Associations between mitochondria and the ER were depicted by both traditional EM and electron tomography (ET). Based on an examination with ET, researchers found that mitochondria and the ER are connected by tethers [26]. These linkages were shown to have a length of 10 nm (for smooth ER) and 25 nm (rough ER). The number of tethers can control the level of communication between interacting organelles [27]. For example, studies by Kannan and Lahiri on yeast determined that connections between mitochondria and the ER could be increased by expression of a ChiMERAconstruct, which is a synthetic ER-mitochondria tether [10,19]. EM images revealed that the average length of an interaction interface between mitochondria and the ER was 250 nm in wild-type cells and 800 nm in cells with ChiMERA expression. An increased amount of MERCs enhanced the transfer of phosphatidylserine (PS) to mitochondria, indicating the importance of MERCs in phospholipid (PL) exchange [19,32]. Other studies on *Saccharomyces cerevisiae* concluded that a small GTPase, Sar1, is responsible for the regulation of the extent of contact sites. Micrographs of a Sar1D32G mutant revealed increased adjacent regions of mitochondria and the ER spanning as much as one micrometer. At the same time, this mutant negatively influenced mitochondrial fission, likely by reducing the efficiency of the recognition of fission sites by the ER [33].

Other studies also demonstrated extensive numbers of MERCs in cells. However, recognition of tethers was hindered due to particle crowding. Interestingly, tethers between smooth ER and mitochondria in situ have a comparable length of 9–16 nm to the length of the linkage-forming particles found in samples of isolated mitochondria. In regards to rough ER and mitochondria in situ, the minimum length is 20 nm because of the steric hindrance of the ribosomes. Previous research has noted that the heterogeneity of the sizes of tethers may be related to the efficiency of communication between the ER and mitochondria [34]. In budding yeast, tethers are known as the ERMES complex, which is an abbreviation for ER-mitochondria encounter structure. Images of close appositions of mitochondria and the ER in *Saccharomyces cerevisiae* collected by EM and ET show that mitochondria are coiled by the reticulum. In this case, mitochondria may be entirely entwined by the ER, which is also responsible for the initial reduction of the diameter of the fragment of the mitochondrial network marked for fission [18]. Giacomello and Pellegrini’s recent cryo-EM studies also showed that the length and width of the described associations depend on the metabolic state of mice hepatocytes. Under specific conditions, their size may increase from 14–20 nm [1].

### 2.2. Dynamic Properties of Contact Sites

A great deal of information regarding interactions between organelles can be derived from static images obtained using EM high-resolution imaging.

**Figure 1:**
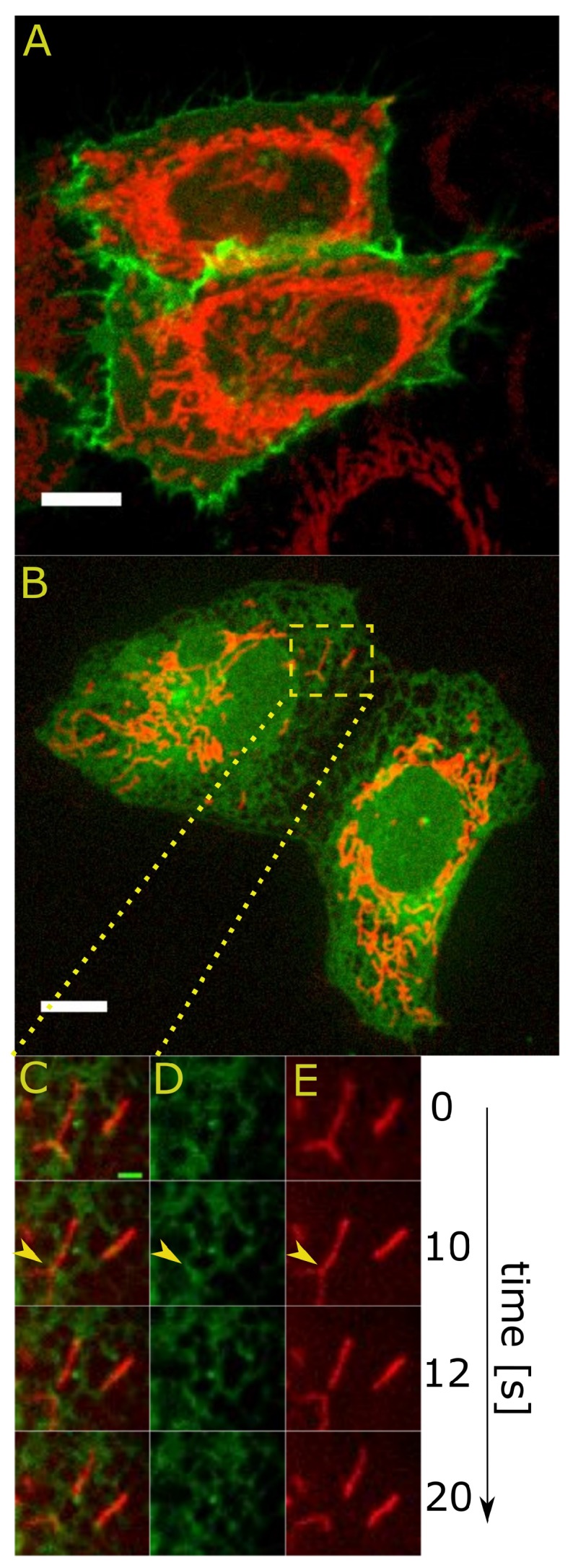
Image of plasma membrane (PM) (green) and mitochondria (red) in HeLa cells. Cells were transfected with pCAG-mGFP (for staining the PM) and mito-mNeptune (for staining the mitochondria). (**A**) Image of the endoplasmic reticulum (ER) (green) and mitochondria (red) in U2OS cells. The cells were transfected with pAc-GFPC1-Sec61-β plasmid (for staining the ER) and mito-mNeptune (for staining the mitochondria); (**B**) An example of a fission event is shown in the enlarged inset region (four selected images from the time series acquired with a 2-s time step). The fission event takes place between the time points of 10 and 12 s. The localization of the fission event is indicated with a yellow arrow in each channel ((**C**) overlay, (**D**) ER, (**E**) mitochondria). The ER structure is clearly visible at the fission site. Imaging was performed using Zeiss spinning disc microscope. The white scale bar in (**A**) and in (**B**) corresponds to 10 μm; the green scale bar in (**C**) corresponds to 2 μm. Experiments were performed with similar procedures as reported elsewhere [18].

However, fluorescence microscopy offers more specificity in imaging than EM since the organelles of interest can be labeled separately using fluorescent proteins (Figure 1) and imaged without seeing the background structures. The several available colors of fluorescent probes provide a way to visualize structures simultaneously, and additional information can be obtained by acquiring dual or tri-color time-lapse series of different structures.

Confocal microscopy in living COS-7 cells showed that ER-mitochondria contact sites colocalize with acetylated microtubules. In their approach, the researchers first analyzed the dynamic nature of interactions between mitochondria and the ER by time-lapse imaging. Obtained confocal images showed that the dynamics of these two organelles is highly interrelated and interconnected and is not disrupted during the movement of both organelles. The researchers also found that every analyzed mitochondrion in the peripheral region of COS-7 cells localized to the ER. After live cell imaging (on gridded coverslips), cells were fixed and immunolabeled for α-tubulin and acetylated microtubules. Re-analysis of already imaged regions in living cells following cell fixation and immunolabeling displayed mitochondrial colocalization with mostly acetylated microtubules. Colocalization was observed for acetylated microtubules with static as well as with dynamic mitochondria. In conclusion, the authors hypothesized that ER dynamics on acetylated microtubules helps to establish and/or maintain contacts between the ER and mitochondria [35].

#### 2.2.1. Fission at the ER-Mitochondria Contact Sites

The studies using live cell imaging revealed that ER marks the sites of mitochondrial fission [18]. Previous experiments showed that the vast majority of analyzed mitochondrial fission events occurred at MERCs in both yeast and mammalian cells. Imaging of COS-7 cells revealed that ER tubules crossover mitochondria in future fission sites, and in these sites, the diameter of mitochondria is narrower than normal. The ER has been suggested to cause initial constriction of mitochondria, which then enables the assembly of a functional Drp1 ring on mitochondria that finally leads to mitochondrial fission [18]. Further investigation of ER-associated mitochondrial fission events with the use of confocal microscopy in living cells showed that this process occurs in proximity to sites of mtDNA replication, which facilitates the uniform distribution of the newly-synthesized mitochondrial genome to daughter mitochondria. This observation was possible due to simultaneous imaging of mitochondria, replicating mitochondrial nucleoids and the ER in living U2OS cells. This finding notes the importance of ER-mitochondria contacts for proper mtDNA maintenance [36].

#### 2.2.2. Joining Forces: An Alliance of Different Detection Methods

Confocal microscopy often adds extra information to the results obtained with EM [18]. Another example of the combination of these two methods is the investigation of the influence of Sar1 on ER-membrane contact sites. The results acquired with EM were already described here, but confocal microscopy imaging was also used to obtain supplementary data concerning this issue. Mutated Sar1 induces alterations in ER morphology in yeast, as well as in mammalian cells (the mammalian homolog is SAR1A), which results in increased length of contact sites between the ER and mitochondria. Thus, Sar1 is a negative regulator of the size of MERCs. Knock down of SARA1 in HeLa cells caused abnormalities in mitochondrial morphology, which were observed in fixed cells via confocal microscopy imaging. This result confirms that Sar1 localized in the ER is involved in the regulation of ER-mitochondria contact sites by which this protein can affect the morphology of the mitochondrial network [33].

Another approach to study contacts between the ER and mitochondria is the combination of confocal live cell imaging with correlative cryogenic fluorescence microscopy and soft X-ray tomography. In experiments conducted with these techniques, the interplay between the ER, mitochondria and MiD proteins was shown during mitochondrial fission. Live cell imaging experiments of COS-7 cells revealed the presence of MiD proteins at MERCs, but only approximately 40% of the observed MERCs at MiD spots were localized to mitochondrial constriction sites [37]. Using cryogenic fluorescence microscopy together with soft X-ray tomography, researchers created a high-resolution 3D model of contacts between MiD proteins and the ER at mitochondria fission sites in v-Abl-transformed lymphoma B-cells. The result showed that at MiD foci, the ER is connected with mitochondria via short, protruding extensions, which have a diameter of 80 nm and a length of approximately 170 nm. MiD51-GFP foci were present at every spot where ER extensions contacted mitochondria. However, the authors of the research took into consideration that what they perceived as ER extensions may also be very thick or highly absorbent actin bundles, as actin polymerization is also engaged in the mitochondrial fission process [37].

#### 2.2.3. Visualization of Contact Sites Based on Artificial Molecular Tethers

The contact sites could be detected using fluorescent proteins (FP) targeted to the different cell compartments. If the two compartments are in contact, the signals from FP would overlap, reporting the presence of contact sites. However, there also fluorescent reporters that enable more selective highlighting of only the structures forming the contact site, which are based on artificial molecular tethers. Such molecules carry two different targeting sequences, which provide the affinity to the respective organelles. The localization of the tether serves as a reporter of the sites of inter-organelle spatial proximity.

A recently introduced group of dimerization-dependent fluorescent proteins (ddFP) enables specific visualization of the dynamics of contact sites between the ER and mitochondria in living cells. Dimerization-dependent proteins work based on the principle that the fluorescence signal in the cell is obtained only when two dark FP monomers (ddFP-A and ddFP-B) exist in this cell close enough to form a fluorescent heterodimeric complex. Therefore, using ddFP enables visualization of spots in the cell in which two proteins of interest interact with each other. To visualize MERCs in a living cell, the authors used the following constructs: ddGFP-A fused to ER protein calnexin and ddGFP-B fused to mitochondrial protein Tom20 [38]. Cotransfection of HeLa cells with these two constructs resulted in green fluorescence mostly in the perinuclear region of cells. Additional cotransfections with mCherry fused to proteins localized to mitochondria or the ER revealed that green fluorescence arising from the interaction of calnexin-ddGFP-A with Tom20-ddGFP-B colocalized with mitochondria to a certain degree, as well as with the ER. This finding showed that the green fluorescence signal was specific and depicted sites of interactions between the ER and mitochondria, which makes the ddFP system a very useful tool in the analysis of contact sites between these two organelles in living cells and allows for the study of changes in this interaction with different aspects and conditions [38].

Another method of imaging MERCs is based on drug-inducible fluorescent interorganelle linkers, which allows one to track connections between the ER and mitochondria in living cells. Apart from simple visualization of these sites, it also enables monitoring of changes in the concentration of Ca${}^{2+}$ in these interfaces. The constructs used in the method are CFP-FRB-ER (colocalized to ER) and OMM-FKBP-FP (colocalized to mitochondria). Addition of rapamycin to RBL-2H3 and H9c2 cells cotransfected with CFP-FRB-ER and OMM-FKBP-FP induces binding of these two constructs at ER-mitochondria contact sites due to the high affinity of rapamycin for FRB (FKBP-rapamycin binding domain, 100-amino acid domain of mTOR) and FKBP (FK506 binding protein), which makes this compound a bridge that joins these two proteins [39,40]. This finding indicates that heterodimerization between FRB and FKBP upon rapamycin treatment should occur only locally in places of proximity between ER and mitochondria. A similar approach has been used to look for contact sites between the PM and mitochondria, but the results of the experiment showed that a relatively large fraction of mitochondria in the perinuclear region of cells is not connected to the plasma membrane. To further study MERCs, the authors applied the FRET method also using constructs that interact after rapamycin treatment. This experiment showed the focal characteristic of the interaction site between the ER and mitochondria. To evaluate the concentration of Ca${}^{2+}$ at ER-mitochondria interfaces, a ratiometric pericam was used, which is a Ca${}^{2+}$-sensitive fluorescent protein. Addition of rapamycin to the cells caused formation of an OMM-pericam-ER complex at MERCs. This study revealed the existence of a microdomain with a high concentration of cytosolic Ca${}^{2+}$ (high [Ca${}^{2+}$]${}_{c}$) at the interface between the ER and mitochondria. This finding indicates that separation between these organelles is needed for efficient calcium delivery [39].

#### 2.2.4. Getting Closer: Super-Resolved Imaging

A super resolution (below 50 nm) method was used to study mitochondria, ER dynamics with 2 s temporal resolution using a specific stimulated depletion microscopy-compatible labeling scheme. Up to 3-min movies were collected, which is sufficiently long for the future detection of interesting cellular events (e.g., fission events). On the images, the OMM was clearly distinguishable from the matrix, and in the case of the ER, the ER lumen could also be distinguished from its membrane. The study did not focus on the detailed analysis of MERCs, but shows that time-lapse imaging of MERCs is possible with high spatial (below 50 nm) and good temporal resolution in live cells, and more quantitative data are on the way [41].

#### 2.2.5. Mobility and Concentrations: Fluorescence Correlation Methods

Live cell imaging of ER-mitochondria contact sites provided a very valuable insight into cellular dynamics; however, often only a fraction of available information was extracted from the time lapse data, focusing on rather isolated single events captured by the human eye. Quite often, microscopy time-lapse data are very complex, making their analysis a challenging task. The whole family of methods called image correlation spectroscopy (ICS) can provide useful information with respect to the description of the dynamic nature of the studied system. These methods originated from fluorescence correlation spectroscopy [42] and can be applied to derive quantitative molecular parameters from microscopy images, such as the diffusion coefficients or concentrations of biological molecules in living cells [43,44]. Different variants of ICS were proposed [45], which address different temporal regimes of dynamic processes. Usually, the application of such methods was restricted to regular (rectangular) and uniformly-bright regions of the image, which hampered its use in more complex images of biological specimens. Despite this limitation, in a number studies, ICS methods were successfully applied in living cells to obtain quantitative data [46]. Using arbitrary raster image correlation spectroscopy, the dynamics of selected organelles can be studied, which paves the way for cross-correlation analysis to follow the dynamics of contact sites between different organelles [47,48]. Such methods of analysis will enable a more automatic method for quantitative estimation of the number and dynamics of membrane contact sites. The rapid development of imaging techniques creates various opportunities for studies of cell dynamics, including interorganelle contact sites.

## 3. Proteins Localized to MAM and Their Role in Calcium Homeostasis

**Figure 2:**
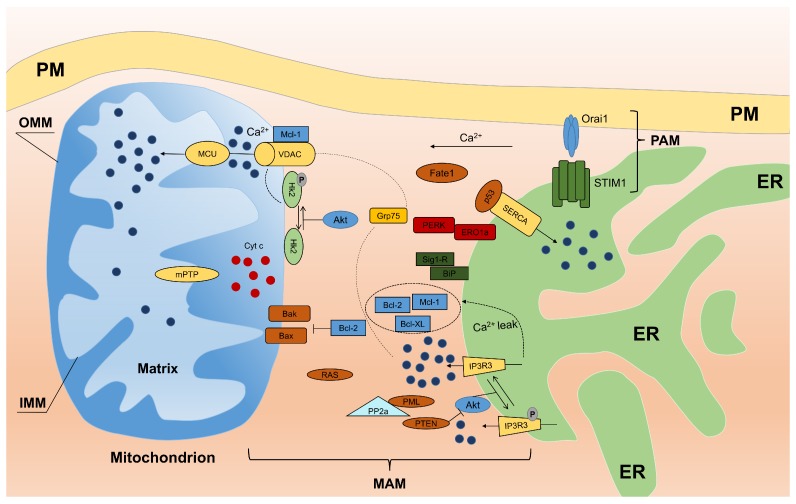
Proteins involved in Ca${}^{2+}$ homeostasis at MAM and PAM fractions. Bak, Bcl-2 antagonist/killer; Bax, Bcl-2 associated X protein; Bcl-2, B-cell CLL/lymphoma 2; BIP1, or GRP78, glucose regulated protein 78; cyt. c, cytochrome c; ER, endoplasmic reticulum; GRP75, glucose regulated protein 75; HK2, hexokinase 2; IP3R, inositol 1,4,5 trisphosphate receptor; MAM, mitochondria associated membranes; Mcl-1, myeloid cell leukemia sequence 1; MCU, mitochondrial calcium uniporter; mPTP, mitochondrial permeability transition pore; Orai1, ORAI Calcium Release-Activated Calcium Modulator 1; PAM, plasma membrane associated membranes; PM, plasma membrane; PML, promyelocytic leukemia protein; PTEN, phosphatase and tensin homolog deleted on chromosome 10; SERCA, sarco/endoplasmatic reticulum Ca${}^{2+}$ ATPase; Sig1R, Sigma 1 receptor; STIM1, Stromal interaction molecule 1; VDAC, voltage-dependent anion channel.

Based on recent reports, proteomic studies of the MAM fraction revealed a long list of proteins (up to 1212 proteins) [4]. However, most of the identified proteins were contaminants. The most likely number of MAM-specific proteins seems to be more than 75 based on Raturi and Simmen [31] or 68 based on Hung et al. [49]. Some of these proteins are involved in calcium transfer between mitochondria and the ER (Figure 2). More about proteins localized in MAM and their function can be found in the paper of Giorgi et al. [50].

### 3.1. Ca${}^{2+}$ Trafficking and Apoptosis

The ER and mitochondria play central roles in the transmission of Ca${}^{2+}$ signals in physiological and pathological conditions where MAM are specialized microdomains for Ca${}^{2+}$ transfer. The ER has been considered the major Ca${}^{2+}$ store inside the cell. The sarco/endoplasmic reticulum Ca${}^{2+}$ ATPase (SERCA) is a protein that transports calcium ions from the cytoplasm into the ER and is enriched at the MAM. The overexpression of SERCA increases spontaneous and induced apoptosis via ER (Ca${}^{2+}$) overload [51,52], favoring ER-mitochondria Ca${}^{2+}$ transfer that triggers defective mitochondrial function. Furthermore, the release of Ca${}^{2+}$ ions from the ER by the transmembrane protein inositol 1,4,5-trisphosphate receptor (IP3R) contributes to the rapid increase in intracellular Ca${}^{2+}$, and thus, the mitochondria take up large amounts of Ca${}^{2+}$ through close contacts with the ER. The ER protein sigma-1 receptor (Sig1R) is also localized to MAM and can also be considered an MAM marker [53]. Sig1R forms a Ca${}^{2+}$ sensitive chaperone complex with BiP (also known as GRP78) and prolongs Ca${}^{2+}$ signaling from the ER to mitochondria by stabilizing type 3 IP3R [54]. Ca${}^{2+}$ efflux from the ER crosses the freely permeable OMM through VDAC channels, reaches the inner mitochondrial membrane (IMM) and accumulates in the matrix by an mitochondrial calcium uniporter (MCU) complex through local microdomains at high [Ca${}^{2+}$], which overtake the apparently low Ca${}^{2+}$ affinity of MCU [55,56]. The MAM chaperone glucose-regulated protein 75 (GRP75) links Ca${}^{2+}$ efflux from ER with VDAC1 at the OMM to regulate Ca${}^{2+}$ uptake at mitochondria. However, excessive accumulation of Ca${}^{2+}$ can activate the release of pro-apoptotic factors, such as cytochrome c and SMAC/DIABLO, into the cytosol due to the opening of mitochondrial permeability transition pore (mPTP) [57,58].

### 3.2. Calcium and Reactive Oxygen Species at MAM

Considering the important function played by mitochondrial Ca${}^{2+}$ in the modulation of various responses, such as apoptosis, numerous factors could be involved in regulation of the size and the number of ER-mitochondria contact sites to manage Ca${}^{2+}$ trafficking. For example, the interactions between the ER and mitochondria are modulated in different ways and by numerous proteins, including the mitochondria-shaping proteins MFN-1/-2 (mitofusin-1/-2). The loss of MFN-2 alters Ca${}^{2+}$ signaling and reduces ER-mitochondria contacts due to MFN-2-induced changes to ER morphology [59]. However, Filadi and colleagues demonstrated that MFN-2 reduces the number of close contact sites between the two organelles and proposed a new model for ER–mitochondria tethering [60].

Moreover, the MAM appears to be a heavily involved domain in the process of ER stress-mediated apoptosis. Among others, mitochondria and ER are two intracellular sites of reactive oxygen species (ROS) production where MAM are the place where ROS exchange can occur. Thus, unsurprisingly, many regulators of the oxidative state of cells are located at ER-mitochondria junctions, which is the case for p66Shc, a cytosolic adaptor protein involved in the cellular response to oxidative stress. The upregulation of ROS production by androgen or estrogens is accompanied by an increase in p66Shc, promoting cell proliferation in prostate cancer cells [61]. The levels of p66Shc in the MAM increase in an age-dependent manner in correlation with mitochondrial ROS production, which has been demonstrated to increase with age [62]. The RNA-dependent protein kinase (PKR)-like ER kinase (PERK) is a key ER stress sensor in the unfolded protein response (UPR) and is enriched at MAM. Here, PERK promotes efficient crosstalk between the ER and mitochondria through Ca${}^{2+}$ and ROS transfer and is crucial for maintaining MERCs and thus for MAM integrity. Cells deficient in PERK display disturbed ER morphology and Ca${}^{2+}$ homeostasis and reduced ROS transfer from the ER to mitochondria [63]. Recent research has shown that Ero1-α (ER oxidoreductin-1 α) is located at MAM where it modulates Ca${}^{2+}$ homeostasis. Ero1-α is a key controller of oxidative folding and ER redox state. The overexpression of redox-active Ero1-α increases passive Ca${}^{2+}$ efflux from the ER to mitochondria in response to IP3R agonists [64].

### 3.3. Oncogenes Link MAM and Calcium Homeostasis

MAM have also been demonstrated to represent a platform for several oncogenes and tumor-suppressors that are listed below:

- PTEN: Phosphatase and tensin homolog deleted on chromosome 10 (PTEN) is one of the most commonly-lost or mutated onco-suppressors in human cancers and is a phosphatase that has dual-specific activity for lipids and proteins and is localized to MAM. At MAM, PTEN regulates ER Ca${}^{2+}$ release through type 3 IP3R in a protein phosphatase-dependent manner that counteracts Akt phosphorylation of type 3 IP3R [65]. Thus, sustained activity of type 3 IP3R enhances Ca${}^{2+}$ transfer from the ER to mitochondria during apoptotic stimulation.
- PML: The tumor suppressor PML (promyelocytic leukemia protein) is localized to MAM. Despite its activity at the nucleus, where PML forms subnuclear structures called PML nuclear bodies, and the cytosol, PML has been shown to localize to the ER and mitochondria [66,67]. Here, PML forms a super-complex with type 3 IP3R, Akt and protein phosphatase PP2A, which regulates Ca${}^{2+}$ and apoptosis. The loss of PML reduces PP2A activity at the ER, leading to Akt activation and type 3 IP3R hyperphosphorylation that inhibits Ca${}^{2+}$ transfer from the ER to mitochondria and consequent apoptosis. In addition, as shown in the study by Missiroli et al. [68], PML localized to MAM is a crucial element not only for apoptosis control, but also for autophagy control in a Ca${}^{2+}$-dependent manner through the AMPK/mTOR/Ulk1 pathway. The reintroduction of MCU in PML${}^{-/-}$ cells increases the ability of mitochondria to accumulate Ca${}^{2+}$ and is sufficient to repress autophagy by reducing the amount of activated AMPK. These data suggest that PML controls autophagy at MAM by exerting its effects on Ca${}^{2+}$ homeostasis.
- p53: Another example of an MAM resident protein is tumor suppressor p53 that regulates tumorigenesis in a Ca${}^{2+}$-dependent pathway in addition to its transcriptional activity. In fact, p53 was recently shown to localize to the ER and MAM compartments where it interacts with SERCA pumps, augmenting Ca${}^{2+}$ release from the ER and the consequent apoptotic program [69]. Furthermore, Giorgi and colleagues demonstrated that extra-nuclear p53 promotion of pro-apoptotic Ca${}^{2+}$ signaling at the ER-mitochondria is important not only for chemotherapy but also for the cellular response following photodynamic therapy [70].
- Bcl-2: Among the members (oncoproteins) of the Bcl-2 family, the “patriarch” is Bcl-2, which is highly enriched at MAM. Bcl-2 exerts its anti-apoptotic function both at the ER and mitochondria. At the ER, Bcl-2 decreases Ca${}^{2+}$ release to mitochondria, which inhibits apoptosis [71,72]. At the mitochondria, Bcl-2 binds Bax/Bak, preventing their oligomerization and Bax/Bak pore formation [73]. Interestingly, as mentioned above, Sig1R regulates Bcl-2 expression in a transcriptional manner by regulating the ROS/NF-κB pathway [74].
- Akt: A serine/threonine kinase (Akt) plays a pivotal role at the ER-mitochondria interface. Akt phosphorylates all IP3R isoforms [75,76,77], inhibits Ca${}^{2+}$ release from the ER and protects cells from apoptosis. Our group showed that Akt inhibits Ca${}^{2+}$ efflux from the ER by preferentially phosphorylating isoform 3 of IP3R [78]. Akt phosphorylates several proteins, including members of the Bcl-2 family (activating their anti-apoptotic properties) as well as hexokinase 2 (HK2). Following phosphorylation by Akt, HK2 binds to VDAC1, inhibiting Ca${}^{2+}$-dependent opening of mPTP and the release of pro-apoptotic factors [79]. A similar activity of Bcl-2 has been described for other members of the family. For instance, Bcl-xL interacts with IP3Rs, decreasing ER Ca${}^{2+}$ concentrations and stimulating mitochondrial energy [80]. Using Bcl-xL knock down cell lines in which ER- and mitochondria-targeted chimeras are reintroduced, Li et al. demonstrated that ER-targeted Bcl-xL is necessary to restore Ca${}^{2+}$ homeostasis, while mitochondrial localization is sufficient to provide protection [81]. Moreover, myeloid cell leukemia protein 1-long isoform (Mcl-1L) is localized to the mitochondrial membrane. This protein controls different processes in mitochondria during apoptosis to counteract the activity of the pro-apoptotic proteins Bak and Bax and to enhance the crucial role of Ca${}^{2+}$ leakage [82].
- H-Ras: Another oncogene, H-Ras, has been shown to localize to both, MAM and PAM. Rimessi et al. showed that Ca${}^{2+}$ signaling has a fundamental role in tumor formation and maintenance promoted by compartmentalized H-Ras [83]. Moreover, oncogenic K-RAS inhibits Ca${}^{2+}$ release from the ER, reducing ER Ca${}^{2+}$ levels and suppressing Ca${}^{2+}$ influx into mitochondria, as observed in colon cancer cell lines [84]. Thus, multiple forms of Ras have an important role at the ER-mitochondria interface in Ca${}^{2+}$ transfer, which contributes to the oncogenic characteristic of Ras.
- FATE1: Fairly recently, fetal and adult testis expressed (FATE1) protein overexpressed in a variety of cancers, was found to be localized to MAM. FATE1 is involved in regulating ER-mitochondria distance, Ca${}^{2+}$ uptake by mitochondria and drug-dependent apoptosis in cancer cells [85].

## 4. MAM: The Lipid Point of View

Since MAM remain under close juxtaposition between the ER and mitochondria, it soon became very clear that they act as a double-edged sword during intercommunication between these organelles via lipids. In fact, MAM not only control lipid-dependent mitochondrial physiology and ER homeostasis but also support direct and functional transition of different lipid species, with relevant biological consequences for cell fate [86]. In this chapter, we discuss the structural and functional features of MAM in lipid trafficking, and we also focus on an emerging role of altered MAM in neuronal and metabolic pathologies.

### 4.1. MAM: A Specific Hub for Lipid Turnover Enzymes

The convenient apposition between the ER/MAM and mitochondria is reversible and does not include membrane fusion [87]. Moreover, MAM seem to be extremely flexible in recruiting specific proteomes at the MAM interface to modulate their formation and function [86]. The outstanding heterogeneity of the MAM structure requires very unique protein and lipid constituents in order to support formation of the MAM interconnection in response to specific cellular conditions. Therefore, MAM were reported to be fortified with cholesterol and sphingolipids, which increase their thickness [88,89]. MAM have also a different degree of curvature, composition of PLs and degree of unsaturation than the surrounding ER membrane [89]. In comparison to the bulk of the ER, mammalian MAM accommodate diverse enzymes involved in lipid trafficking and synthesis, including acyl-CoA: cholesterol acyltransferase/sterol *O-*acyltransferase 1 (ACAT1/SOAT1), diacylglycerol *O-*acyltransferase 2 (DGAT2), PS synthases 1 and 2 (PSS1 and PSS2), phosphatidylethanolamine *N-*methyltransferase 2 (PEMT2) and fatty acid CoA ligase 4 (FACL4/ACS4) [90,91,92,93,94]. ACS4 activates long chain fatty acids that will be used for the synthesis of complex lipids or acylated proteins. Of the five ACS isoforms, only ACS1 and ACS4 localize to MAM [95]. Interestingly, targeting of particular proteins to the MAM requires a mitochondrial targeting signal, which was found in the N-terminal region of DGAT2 [91], or depends on the target’s palmitoylation status as observed with calnexin and thioredoxin 1 [96]. DGAT2 catalyzes the final step of triacylglycerol synthesis [91], and it was reported to colocalize with stearoyl-CoA desaturase 1 (SCD1) in the ER [97]. Remarkably, SCD1, which is the rate-limiting enzyme during the biosynthesis of monounsaturated fatty acids from saturated substrates, specifically palmitate and stearate [98], was also enriched in MAM [97]. Moreover, diminished SCD1 activity was associated with altered accumulation, composition, and saturation status of cellular membrane PLs and neutral lipids [99].

### 4.2. Role of ER-Mitochondria Connectivity in Lipid Synthesis and Transport

**Figure 3:**
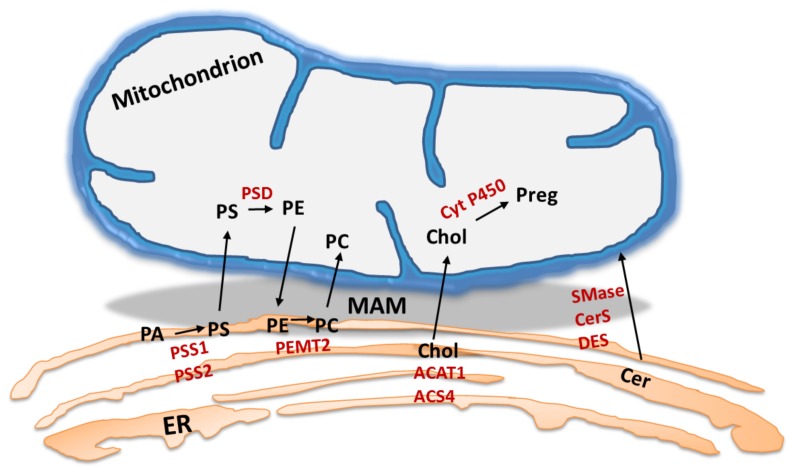
Lipid trafficking at the mitochondria-associated membranes. The ER-mitochondria interface fosters the transport of phospholipids, cholesterol (Chol) and ceramides (Cer). Particular lipid species exodus is integrated by a network of MAM residing enzymes (marked in red), including PSS1, PSS2, PSD, PEMT2, cytochrome P450, SMase, CerS and DES. Abbreviations: PA, phosphatidic acid; PS, phosphatidylserine; PE, phosphatidylethanolamine; PC, phosphatidylcholine; Preg, pregnolone.

MAM were initially described as domains enriched in PL enzymes [100]. Indeed, PL synthesis is usually restricted to the ER; however, PL needs to be subsequently translocated to other organelle membranes (Figure 3). PS is synthesized in the ER by the MAM enzymes, PSS1 and PSS2. After translocation to the mitochondria, PS decarboxylase (PSD) converts PS into phosphatidylethanolamine (PE). Finally, PE returns to the ER, where PEMT2 methylates it for the synthesis of phosphatidylcholine (PC). Both enzymes, PSS1 and PSS2, catalyze serine exchange activity, whereas PSS1 fosters choline exchange [87,101]. The rate-limiting step in PE generation is the transfer of PS into mitochondria through the MAM [87]. The transport of PS was dependent on the PS-acyl chain composition in HeLa cells, particularly the 38:4 and 38:5 species were imported more efficiently. In contrast, the corresponding species of PE were exported from mitochondria with less effort [102]. Inhibition of PSD activity with hydroxylamine or depletion of cellular ATP resulted in accumulation of PS in the MAM of CHO cells [103].

In addition, MAM contain enzymes necessary for cholesterol and ceramide biosynthesis (Figure 3) [3,104,105,106]. Genetic deficiency of the key regulator of cholesterol efflux, caveolin-1, in hepatic MAM led to reduced MAM physical extension and integrity, as well as aberrant free cholesterol accumulation [107]. In the basal or resting state, ACAT1, which is located in the MAM, catalyzes the formation of cholesteryl esters from free cholesterol, thereby controlling the equilibrium between membrane-bound and cytoplasmic lipid droplet-stored cholesterol [108]. Under a stress response, cholesterol import to mitochondria is sustained, and cytochrome P450 subsequently initiates steroidogenesis [3]. In acute stress or hormonal stimulation, MAM-associated steroidogenic acute regulatory (StAR) protein fosters cholesterol into mitochondria via critical interaction with VDAC2, thereby initiating mitochondrial steroidogenesis [109]. Additionally, the AAA domain-containing protein 3 (ATAD3) was enriched in the MAM of MA-10 cells, and it is believed to participate in the regulation of steroidogenesis by MAM formation, thereby channeling cholesterol between the ER and mitochondria [106].

It is tempting to speculate that a high concentration of cholesterol in lipid raft microdomains of MAM could deliver free cholesterol for steroidogenesis in mitochondria [87,110]. Depletion of cholesterol or inhibition of de novo synthesis of ceramides in CHO cells drives the relocation of MAM proteins, such as Sig1R and IP3 receptors, towards ER cisternae [110]. Moreover, lowering cholesterol in MAM significantly promoted the association between MAM and mitochondria and led to a decline in de novo synthesis of PS with simultaneous improvement of PE synthesis [3].

A certain pool of the ceramides appears to be produced in MAM since they contain sphingomyelin phosphodiesterase (SMase), ceramide synthase (CerS) and dihydroceramide desaturase (DES) in the proteome (Figure 3) [104,105,111]. Considering the proapoptotic identity of ceramides at mitochondria, MAM might represent a critical reservoir or barrier to prevent ceramide influx.

## 5. Consequences of MAM Dysfunction and MAM Lipid Metabolism Defects

Even though the protein profile is critical for ER-mitochondria connectivity, the lipid composition is responsible for its recruitment at the MAM location and proper activity. Defects in lipid metabolism, resulting in incorrect assembly and functioning of MAM-mitochondria contact sites, are likely to reflect the onset and progression of various human neurodegenerative and metabolic disorders.

### 5.1. MAM Collapse in Amyotrophic Lateral Sclerosis

Recent studies clearly show that the collapse of MERCs is an important mechanism involved in ALS pathology, where abnormalities in MAM interfere with calcium homeostasis, ER, mitochondria, and oxidative stress [112,113,114,115].

The causes of ALS are numerous, and the mechanism of the disease remains far from clear. ALS is characterized by rapid degeneration of motor neurons in the spinal cord, which leads to aggressive progression of muscle paralysis and death within a few years of diagnosis [116]. The mean age of onset of ALS is between 50 and 65 years [116]. Hence, an advanced age is a major risk factor for the development of ALS and may be due to an age-associated reduction of motor neurons, leading to sarcopenia [117]. The median incidence rate of ALS is approximately 2/100,000, and only approximately 10% of ALS cases are inherited (familial, fALS) [118]. Several genetic abnormalities are associated with ALS. The dominant mutation in Cu/Zn superoxide dismutase (SOD1) is one of the most frequent causes of fALS [113,119,120]. Other risk factors, including oxidative stress, reduced rate of autophagy, RNA toxicity, loss of protein homeostasis, and also, several neuro-related abberations like excitotoxicity, neuroinflammation, defective axonal transport were connected with pathophysiology of ALS [121,122,123,124].

Mitochondrial abnormalities are common in many forms of ALS and include morphological and functional aberrations, such as disruption of Ca${}^{2+}$ homeostasis, transport and bioenergetics, and ROS overproduction. Additionally, ER stress and changes in ER protein function and mislocalization are associated with ALS [125]. Recently, MAM “collapse” was also implicated in onset and progression of the disease [112]. Disruption of ER-mitochondria contact sites has been found in SOD1-, Sig1R-, TDP-43-, and FUS-related ALS [112,126,127]. The studies focused on the involvement of these proteins in ALS pathogenesis are summarized below:

- Sig1R, a gene product of *SIGMAR1*, is a chaperone protein expressed in spinal cord [128]. As mentioned previously, Sig1R is localized to MAM and is involved in lipid export and calcium signaling by acting as a ligand-operated receptor chaperone for type 3 IP3R [54]. Mutations in the *SIGMAR1* gene cause a juvenile form of ALS (ALS16) [129]. Prause et al. [130] showed the reduced Sig1R levels in the spinal cords of ALS patients. Moreover, Sig1R KO mice exhibited locomotor deficits associated with muscle weakness, axonal degeneration and motor neuron loss. The lack of Sig1R in motor neurons disturbed MERCs, affected intracellular calcium signaling and induced ER stress. Consequently, loss of Sig1R affects mitochondrial dynamics and transport. Intracellular calcium scavenging and inhibition of ER stress restored mitochondrial function and consequently prevented motor neuron degeneration [131]. Furthermore, non-functional Sig1R variants responsible for the inherited juvenile ALS16 or Sig1R deficiency in transgenic SOD1-linked ALS mouse model were associated with impaired ultrastructure of the MAM, depletion of the Sig1R interacting partners at the MAM and deregulation of calcium homeostasis via mislocalization of the MAM-residing IP3R [112]. Consequently, disruption of MERCs in Sig1R-depleted cells was associated with lipid raft alterations and defective endolysosomal pathways [132]. The number of ER-mitochondria contact sites was decreased in Sig1R-depleted cells and in cells accumulating mutated SOD-1 by 7.5 and 8.2%, respectively [112].
- VAPB integral ER protein. VAPB protein is also enriched in MAM and interacts with the mitochondrial outer membrane protein, tyrosine phosphatase-interacting protein-51. This interaction is critical for the maintenance of MERCs [126]. Deprivation of either of these two constituents results in the loss of the ER-mitochondria interconnection and defects in mitochondrial calcium uptake. Nishimura et al. [133] revealed that a mutation in VAPB causes late-onset spinal muscular atrophy and ALS. Moreover, similar to Sig1R, reduced VAPB expression in the spinal cord has been reported in sporadic ALS, suggesting the impairment of ER/mitochondria contacts and the UPR [134]. Interestingly, Kim et al. [135] showed that neuronal overexpression of wild-type human VAPB slows disease progression and increases survival in SOD1G93A transgenic mice.
- SOD1. Although SOD1 is not a MAM protein, it plays an important role in MAM functioning [113]. The association between SOD1 mutation and MAM function can be confirmed by abnormal calcium release from the ER in astrocytes derived from SOD1 mutant mice [136]. Interestingly, in the spinal cord, mutated SOD1 binds to the outer mitochondrial membrane (OMM) [137] but also accumulates in the MAM fraction. Watanabe et al. [112] showed that association of mutated SOD1 with the MAM prevents the association of the OMM with the ER. Previous research has also shown that MAM protein, mitochondrial E3 ubiquitin ligase MARCH5 also known as MITOL, ubiquitinates and targets mutated SOD1 for degradation and that autophagosome formation is initiated at the MAM [112,138,139]. The relationship between dysfunctional proteostasis and ALS is also evident in the case of mutations in the ATPase VCP (the cause of 1–2% of fALS cases) [140,141,142].
- VCP is involved in ER-associated protein degradation, ER stress and autophagy [143]. Mutated VCP has been linked to altered TDP-43 metabolism in the spinal cord motor neurons of mutant VCP transgenic mice exhibiting TDP-43 pathology [144]. Thus, the disruption of MERCs induces ER stress and the ER–UPR (an intracellular signaling pathway that is activated by the accumulation of unfolded proteins in the ER) [115], possibly by disturbing the variety of ER chaperones, such as BiP, calnexin, calreticulin, ERp44, ERp57, and the above-mentioned MAM resident Sig1R [54].
- MAM, cholesterol and ALS. Why the free cholesterol content of purified MAM is 7 times higher than that of microsomes still remains unclear [3]. Cholesterol depletion in MAM induced by methyl-β-cyclodextrin significantly increases the association of mitochondria with the ER [3] and influences mitochondrial bioenergetics and structure [145,146]. Moreover, in the spinal cords of ALS patients and in a transgenic mouse model of ALS (Cu/Zn SOD mice), levels of sphingomyelin, ceramides and cholesteryl esters were significantly increased, indicating the possibility of MAM disequilibrium [147]. Defective cholesterol metabolism occurred in ALS and its relevance to MAM requires further investigation.

### 5.2. Alterations in MAM in Alzheimer’s Disease

Disorganization of the ER-mitochondria interface appear to be important in the progression of AD. However, in histological sections of brains of human AD patients, up-regulated MAM-associated proteins were found. In addition, the accumulation of β-amyloid (Aβ) aggregates and overexpression of presenilin-2 elevate the number of ER-mitochondria interactions, favoring Ca${}^{2+}$ transfer between these organelles [148,149]. In AD, excessive amounts of Aβ plaques are deposited in neurons in the brain. Proteolytic cleavage of amyloid precursor protein (APP) by major components of the γ-secretase complex, presenilin-1 and -2, is responsible for generating toxic Aβ peptides [89]. Presenilins and the majority of the γ-secretase activity were found to occur in MAM [150,151]. Furthermore, an increased ER-mitochondria interconnection area was detected in mouse presenilin mutant fibroblasts and fibroblasts of sporadic AD patients or AD individuals with familial mutations in presenilins or APP. Likewise, the higher degree of ER-mitochondria apposition was followed by elevated MAM function, as measured by cholesteryl ester and PL synthesis [2]. Moreover, the up-regulation of MAM–associated proteins, including ACAT1 [2,152], and their activity was reported in human AD brain cortical tissue, APP${}_{Swe/Lon}$ mice, and primary neurons exposed to Aβ [148]. In fact, ACAT1 was reported to be required for APP processing, but the generation of Aβ occurs by an unknown mechanism. Given that the activity of γ-secretase also occurs in the MAM, these proteins might cooperate to maintain a strict intracellular cholesterol balance [89]. Interestingly, tremendous changes in PL species were observed in brain samples of AD individuals [153,154]; however, an elevation of membrane cholesterol, cholesteryl esters, sphingomyelin, and ceramides, which results in increased levels of Aβ, was also reported in cellular systems, AD mouse models and AD patients or cadavers [88,154,155]. The same features of changed MAM function and topology were observed in cases of sporadic AD in which presenilin and APP genes are normal. The ε4 variant of the *APOE* gene encoding apolipoprotein E (ApoE4) carries a higher factor of risk of sporadic cases of AD than ApoE3 [156]. ApoE ferries cholesterol and lipids via high-density lipoproteins, and only astrocytes deliver cholesterol to neurons in the brain. Accordingly, an increase in MAM connectivity and activity was observed in fibroblasts treated with ApoE4-enriched astrocyte-conditioned media compared with those treated with ApoE3 lipoproteins [156]. Thus, this finding fits with the notion that in pathophysiology of familiar or sporadic AD, cholesterol content and its handling governs internalization/processing of APP and activity of the accompanying enzymes at the MAM. Ultimately, these findings correspond to a hypothesis, which states that alterations in MAM function and integrity become hallmarks in the pathogenesis of many neurodegenerative disorders.

### 5.3. MAM Dysfunction in Diabetes Mellitus Type 2

An exciting area of research has emerged due to a few studies showing a connection between aberrant MAM and obesity-related T2DM. Two of the most validated pathophysiological features of T2DM are systemic insulin resistance and pancreatic β cell failure. Particularly, excessive lipid intake or prolonged ectopic fat deposition in tissues (called lipotoxicity) is thought to be the primary reason for islet dysfunction and disease progression [157]. As previously highlighted in this review, the ER-mitochondria interface is a prime determinant of intracellular Ca${}^{2+}$ homeostasis, and proper calcium distribution is crucial for insulin production and secretion by pancreatic β cells. Moreover, MAM are important hubs for insulin signaling due to several proteins that were detected at the MAM location, including protein kinase AKT, mTORC2, and PTEN [158]; however, this interconnection awaits further investigations, particularly in the pancreas. Since T2DM is associated with alterations in lipid metabolism, and ER-mitochondria contact sites foster lipid species exchange between these organelles, one favorable hypothesis is that MAM integrity and actions participate in lipotoxicity in diabetes. In fact, MAM integrity was required for insulin signaling, and it was altered in palmitate-induced insulin resistant HuH7 hepatic cells, as well as in the liver of leptin-deficient ob/ob mice and high-fat and high-sucrose fed mice. Furthermore, disruption of MAM integrity by genetic or pharmacological inhibition of MAM-residing protein cyclophilin D-induced insulin resistance in animals and led to aberrant insulin signaling in human primary hepatocytes. Enhancement of MAM formation restored hepatic insulin signaling and activity in HuH7 cells and diabetic mice [159]. Moreover, an abnormal increase in MAM formation was reported in the livers of ob/ob, and high-fat fed mice, in addition to mitochondrial calcium overload, compromised mitochondrial capacity and augmented oxidative stress [160].

## 6. Summary

An increasing number of publications describing the molecular composition and possible involvement of MAM in different pathologies emphasize the importance of these interactions in cell physiology and pathology. However, due to the nature of the interactions between mitochondria and other organelles (“kiss and run” mode), investigations of their protein and lipid composition is not easy. Even isolation of highly purified MAM and PAM fractions can be fraught with a high risk of nonspecific organelle cross-contamination. Moreover, the isolated MAM fraction cannot be guaranteed to consist of MERCs, including both rough and smooth ER [49]. Ultimately, a combination of microscopy visualization techniques and high-throughput proteomics will shed more light on the molecular structure of the contacts between mitochondria and other organelles, thus improving our understanding of the role of MAM and PAM in cell functioning.

## Abbreviations

(Aβ)	β-amyloid
ACAT1	Acyl-CoA:cholesterol acyltransferase
Akt	Serine/threonine protein kinase
ALS	Amyotrophic lateral sclerosis
Alzheimer’s disease	AD
AMPK	AMP-activated protein kinase
ApoE4	Apolipoprotein E
APP	Amyloid precursor protein
ATAD3	AAA domain-containing protein 3
Bak	Bcl2 antagonist/killer
Bax	Bcl-2 associated X protein
Bcl-2	B-cell CLL/lymphoma 2
Bcl-xL	Antiapoptotic protein Bcl-xL
BiP	Binding immunoglobulin protein, also known as 78-kDa glucose-regulated protein
CerS	Ceramide synthase
cyt. c	Cytochrome c
ddFP	Dimerization-dependent fluorescent protein
DES	Dihydroceramide synthase
DGAT2	Diacylglycerol O-acyltransferase
Drp1	Dynamin-related protein 1
EM	Electron microscopy
ER	Endoplasmic reticulum
ERMES	ER-mitochondria encounter structure
Ero1-α	ER oxidoreductin-1 α
ERp44	Endoplasmic reticulum resident protein 44
ERp57	Protein disulfide-isomerase A3, also known as 58-kDa glucose-regulated protein
ET	Electron tomography
FACL4/ACS4	Fatty acid CoA ligase 4
FATE1	Fetal and adult testis-expressed transcript protein
FKBP	Rapamycin-binding protein
FP	Fluorescent protein
FRB	FKBP-rapamycin binding domain of mTOR
FRET	Fluorescence recovery after photobleaching
FUS	RNA-binding protein FUS
GRP75	Glucose-regulated protein 75
HK2	Hexokinase 2
H-Ras	GTPase HRas
ICS	Image correlation spectroscopy
IP3	Inositol 1,4,5-trisphosphate
IP3R	Inositol 1,4,5-trisphosphate receptor
K-Ras	GTPase KRas
MAM	Mitochondria-associated membranes
MARCH5/MITOL	E3 ubiquitin-protein ligase MARCH5
Mcl-1	Induced myeloid leukemia cell differentiation protein Mcl-1
Mcl-1L	Mcl-1 long isoform
MCU	Mitochondrial calcium uniporter
Mdm36	Mitochondrial distribution and morphology protein 36
MERC	Mitochondria-ER contacts
MFN	Mitofusin
MiD	Mitochondrial dynamics protein
mPTP	Mitochondrial permeability transition pore
mtDNA	Mitochondrial DNA
mTOR	Mammalian target of rapamycin
NF-κB	Nuclear factor κB
Num1	Nuclear migration protein 1
OMM	Outer mitochondrial membrane
Orai1	ORAI calcium release-activated calcium modulator 1
p53	Tumor protein p53
p66Shc	SHC-Transforming protein 1
PA	Phosphatidic acid
PAM	Plasma membrane-associated membranes
PC	Phosphatidylcholine
PE	Phosphatidylethanolamine
PEMT2	Phosphatidylethanolamine N-methyltransferase 2
PERK	Protein kinase R (PKR)-like ER kinase
PL	Phospholipid
PM	Plasma membrane
PML	Promyelocytic leukemia protein
PP2A	Serine/threonine-protein phosphatase PP2A
Preg	Pregnolone
PS	Phosphatidylserine
PSD	PS decarboxylase
PSS1	PS synthase 1
PSS2	PS synthase 2
PTEN	Phosphatase and tensin homolog deleted on chromosome 10
ROS	Reactive oxygen species
Sar1	Small COPII coat GTPase 1
SARA1	Mammalian homolog of Sar1
SCD1	Stearoyl-CoA desaturase 1 SCD1
SERCA	Sarco/endoplasmatic reticulum calcium ATPase
Sig1R	Sigma non-opioid intracellular receptor 1
SMAC/DIABLO	Second mitochondria-derived activator of caspases/direct IAP-binding protein with low PI
SMase	Sphingomyelin phosphodiesterase
SOAT1	Acyl-CoA:sterol O-acyltransferase
SOC	Store-operated calcium entry
SOD1	Cu/Zn superoxide dismutase
StAR	Steroidogenic acute regulatory protein
STIM1	Stromal interaction molecule 1
T2DM	Diabetes mellitus type 2
TDP-43	TAR DNA-binding protein 43
Tom20	Mitochondrial import receptor subunit TOM20
Ulk1	Unc-51-like kinase 1
UPR	Unfolded protein response
VAPB	Vesicle-associated membrane protein-associated protein B/C
VCP	Transitional endoplasmic reticulum ATPase
VDAC	Voltage-dependent anion channel
VDAC2	Voltage-dependent anion-selective channel protein 2
